# Variability in Prefrontal Hemodynamic Response during Exposure to Repeated Self-Selected Music Excerpts, a Near-Infrared Spectroscopy Study

**DOI:** 10.1371/journal.pone.0122148

**Published:** 2015-04-02

**Authors:** Saba Moghimi, Larissa Schudlo, Tom Chau, Anne-Marie Guerguerian

**Affiliations:** 1 University of Toronto, Toronto, Ontario, Canada; 2 Hospital for Sick Children Research Institute, Toronto, Ontario, Canada; 3 Institute of Biomaterials and Biomedical Engineering, Toronto, Ontario, Canada; 4 Holland Bloorview Kids Rehabilitation Hospital, Toronto, Ontario, Canada; Center for BrainHealth, University of Texas at Dallas, UNITED STATES

## Abstract

Music-induced brain activity modulations in areas involved in emotion regulation may be useful in achieving therapeutic outcomes. Clinical applications of music may involve prolonged or repeated exposures to music. However, the variability of the observed brain activity patterns in repeated exposures to music is not well understood. We hypothesized that multiple exposures to the same music would elicit more consistent activity patterns than exposure to different music. In this study, the temporal and spatial variability of cerebral prefrontal hemodynamic response was investigated across multiple exposures to self-selected musical excerpts in 10 healthy adults. The hemodynamic changes were measured using prefrontal cortex near infrared spectroscopy and represented by instantaneous phase values. Based on spatial and temporal characteristics of these observed hemodynamic changes, we defined a consistency index to represent variability across these domains. The consistency index across repeated exposures to the same piece of music was compared to the consistency index corresponding to prefrontal activity from randomly matched non-identical musical excerpts. Consistency indexes were significantly different for identical versus non-identical musical excerpts when comparing a subset of repetitions. When all four exposures were compared, no significant difference was observed between the consistency indexes of randomly matched non-identical musical excerpts and the consistency index corresponding to repetitions of the same musical excerpts. This observation suggests the existence of only partial consistency between repeated exposures to the same musical excerpt, which may stem from the role of the prefrontal cortex in regulating other cognitive and emotional processes.

## Introduction

Music is capable of modulating brain activity in regions involved in emotional processing [1] (e.g. amygdale, frontal and prefrontal cortex) in addition to brain areas associated with auditory perception. Using music as a means of improving psycho physiological well-being of individuals may be a meaningful pursuit based on the e evidence supporting physiological modulations in response to music [2–3]. To date, music has been used as a clinical intervention in a number of studies. Soto et al. reported enhanced visual awareness in patients with visual neglect when listening to self-selected pleasant musical excerpts [4]. In this study, magnetic resonance imaging (MRI) analysis demonstrated strong functional coupling between orbitofrontal cortex, involved in emotional processing, and posterior parietal cortex associated with attention.

Music-induced activity modulations, observed in areas associated with emotional processing (e.g. amygdale, frontal and prefrontal cortex), has motivated the use of music as an intervention for individuals experiencing anxiety or depression symptoms [5–6]. In a randomized control trial, Chan et al. reported a significant decrease in self-reported depression level due to exposure to music in older adults [7]. In a study by Vachiramon et al., exposure to music during surgery resulted in reduced anxiety levels [8]. Chlan et al. administered patient-directed music during ventilator support, in a clinical trial involving 373 critically ill patients [9]. The results of this study indicated a significant decrease in anxiety score levels and a significant reduction in the intensity and frequency of sedation in the group exposed to music compared to the group receiving usual care.

Administration of music as a clinically proven intervention is still in its infancy and more studies are required in this area [5,7]. One of the important aspects in such studies is the choice of music. Familiarity of musical stimuli may play an important role in the observed effects. Van Den Bosch et al. observed significant changes in the arousal level, measured using electrodermal response, when participants were exposed to unfamiliar music for the first time compared to a second exposure to the same musical excerpt [10]. In a functional MRI study, Pereira et al. observed significantly larger activities in the limbic and paralimbic areas, associated with emotional processing, when exposed to familiar compared to unfamiliar music [11]. In fact, in many studies, self-selected musical excerpts were used [4,7,8]. In prolonged studies involving music, the use of self-selected songs may require repeated exposure to the same musical excerpts. While some have evaluated the effect of patient-directed music interventions, the limited understanding of the mechanisms underlying their therapeutic effects [9] makes it challenging to design most efficacious exposure regimens. The specific effects of repeated exposure to the same music are not well-understood. In this study, the effect of repeated exposure to self-selected musical excerpts was investigated with respect to spatial and temporal hemodynamic response in the prefrontal cortex. The prefrontal cortex is part of the emotional circuitry in the brain. In addition, it can be non-invasively monitored using near-infrared spectroscopy. Near-infrared spectroscopy (NIRS) measures oxygenated and deoxygenated hemoglobin concentrations ([HbO_2_] and [Hb]) and is suited for long-term bedside monitoring in adults, children, and neonates. These characteristics render NIRS plausible for naturalistic studies of emotion-related brain activity. Recent findings have confirmed significant associations between prefrontal hemodynamic response, measured using NIRS, and emotional experience [12–13]. In a study involving pictures with emotional content, Hoshi et al. reported regional changes in [HbO_2_]. Similarly, Tai and Chau were able to differentiate the response to affective pictures from baseline with an average of 75% accuracy, using prefrontal cortex NIRS [14].

Response to music is a dynamic phenomenon. For example, physiological response during the introduction to a musical excerpt may differ from what follows as the music unfolds. In addition to such temporal dynamics, the spatial distribution of the prefrontal hemodynamics is also of great interest in characterizing the response to music. Therefore, in the current study, prefrontal hemodynamic response was compared across multiple exposures to the same self-selected music, by introducing a measure of spatial and temporal consistency. This measure was developed by combining regional phase distinction and spatial moments derived from the regional prefrontal NIRS measurements. Using this measure, the consistency was assessed across four repetitions of the same musical excerpt, and a subset of these repetitions in 10 healthy adults. To the best of our knowledge, the proposed method has not been previously used for fNIRS data analysis and is introduced in this article to meet the specific demands of the research question and investigate if the spatiotemporal characteristics are different across repetitions of the same song.

## Material and Methods

### Procedures

Ten healthy adults were recruited (5 female, age: 25 ± 2.7 years) for this study. The Bloorview Research Institute research ethics board approved of the study and informed written consent was provided by all participants.

Each experimental block comprised 10 seconds of noise followed by 45 seconds of aural stimulus and a final 5 seconds of noise, as depicted in Table 1. In each block, the aural stimulus was either a music excerpt or an emotionally neutral noise recording.

**Table 1:** Experimental block sequence.

Onset beep	Noise	Aural stimulus	Noise	Offset beep	Participant rating
**2 sec.**	**10 sec.**	**45 sec.**	**5 sec.**	**2 sec.**	**N/A**

**Fig 1:**
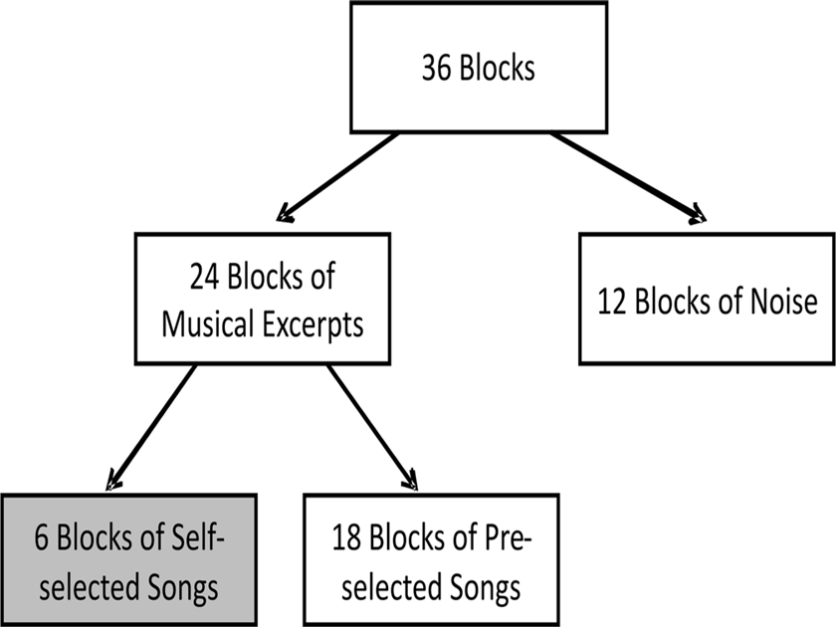
Organization of blocks for each session. 36 blocks were collected during each session. The aural stimulus for 6 of the blocks (marked in grey) consisted of a unique song selected by the participant. Only the 6 blocks of self-selected songs collected from each session were considered in this analysis.

The study was conducted over 4 separate sessions encompassing 36 blocks each (12 noise blocks, 24 musical excerpts). Data collection sessions were conducted separately, such that they did not take place on the same day. Fig. 1 summarizes the experimental blocks during one recording session. The blocks used for the current analysis are shaded in grey.

All experiment sessions were conducted in a dimly lit room. The auditory stimuli/cues were delivered using a headset as the participants sat comfortably in front of a computer screen. The participants were asked to close their eyes after hearing the onset beep and open them after hearing the offset beep to rate their emotions. After each block, participants were prompted to rate their emotions in terms of arousal and valence using a nine level self-assessment manikin [15].

### Stimuli

The music collection used was composed of two subsets: six self-selected songs specific to each participant and 72 music pieces identically played for all participants. In addition, individuals were exposed to randomly distributed blocks with noise as the aural stimuli. The data corresponding to the noise blocks and the 72 common music excerpts were part of another study and were excluded from the current analysis. Therefore, the current investigation was nested in a study involving more musical excerpts and noise blocks. The nested blocks of self-selected musical excerpts were envisioned in the study design and all of the samples corresponding to the self-selected musical excerpts were included for this study to avoid selection bias. The self-selected music excerpts were played once per session. Therefore, each participant was exposed to four repetitions of the same song. Each participant was instructed to select three songs, which induced intense positive emotions, and three, which induced intense negative emotions, prior to the study. Hence a total of 60 blocks were included for the analysis.

### Instrumentation and data preprocessing

**Fig 2:**
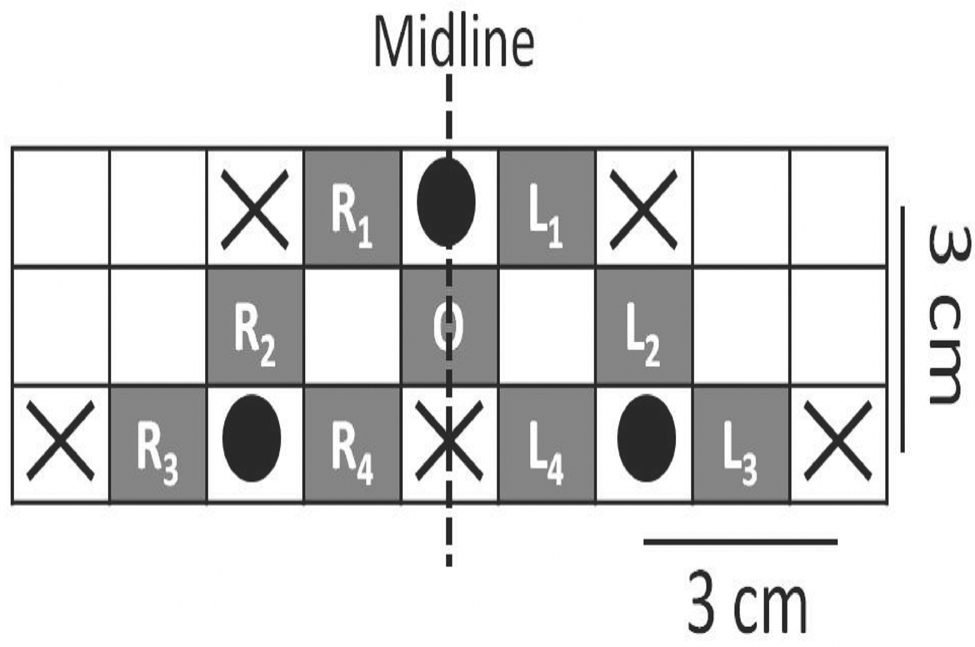
Recording layout. The layout of light sources (circles) and detectors (X's). The vertical line denotes anatomical midline. The annotated shaded areas correspond to recording locations.

An Imagent Functional Brain Imaging System from ISS Inc. (Champaign, IL) was use for NIRS measurements across nine different regions on the forehead, as shown in Fig. 2. Five light sources and three photodetectors were secured to the forehead in a configuration such that each source and its neighbouring detector(s) were 3cm apart. Each source housed two lasers, emitting light at 830nm and 690nm to each location simultaneously. Only measurements obtained from sources and detectors separated by 3cm were considered, resulting in a total of nine measurement points over the anterior prefrontal cortex. The configuration was placed such that the midline was aligned with the participant’s nose and the bottom row of sources and detectors sat just above the person’s eyebrows. Each detector in the bottom row of the configuration sat approximately over the FP1 and FP2 locations of the 10–20 International System. The data were sampled at 31.25Hz. A type II third order Tchebichef low pass filter with a cut-off frequency of 0.1 Hz was used to remove high frequency artifacts such as respiration and heart rate [16]. The 830 nm and 630 nm light intensities at each of the nine recording sites were used to calculate [HbO_2_] and [Hb] by applying the modified Beer–Lambert law [17]. The [Hb] waveforms were used to correct motion-related noise in the [HbO_2_], using the method introduced by Cui et al. [18]. Further analysis was conducted based on the resulting [HbO_2_].

### Data analysis

The hemodynamic changes were compared across the four repetitions by introducing a spatio-temporal measure of consistency. The spatial i.e., regional, patterns of [HbO_2_] were compared by using instantaneous phase. For each recording region shown in Fig. 2, the instantaneous phase (e.g. φ(t)) value was determined using Hilbert’s transform [19].

The phase value of each recording site was compared to the neighboring recording locations, within a 3 cm radius. For example, for the central region O (refer to Fig. 2), L_1_, L_4_, R_1_, and R_4_ were included for the comparison, and for L_3_, regions L_2_ and L_4_ were included for comparison. The comparison was conducted by finding the mean squared error between the neighboring regions (within 3 cm radius) and each recording location. Hence, for the previous example corresponding to L_3_, the mean squared error (MSE_**L3**_) was determined as shown in (1).

$$MSE_{L3}\left(t\right)\text{= }\frac{\text{1}}{\text{2}}\left({\left(\text{[}HbO_{2}\right]}_{L3}\left(t\right)\text{-}{\left(\text{[}HbO_{2}\right]}_{L2}\left(t\right){\text{))}}^{2}\text{+}{\left(\text{[}HbO_{2}\right]}_{L3}\left(t\right)\text{-}{\left(\text{[}HbO_{2}\right]}_{L4}\left(t\right){\text{))}}^{2}\right)$$ (1)

In this example, distinct hemodynamic changes centralized at region L_3_, would result in an increase in the *MSE*_*L3*_(*t*) value. Therefore, *MSE*_*region*_(*t*) was used as a measure of instantaneous phase distinction for each recording region (i.e., center). To emphasize larger distinctions, the MSE was exponentially mapped to the phase distinction waveform (PDW) as shown in (2).

$$PDW_{r}{}_{egion}(t)=e^{MSE_{region}(t)}(2)(whereregion:L1,L2,L3,L4,O,R1,R2,R3,R4)$$ (2)

In this manner, nine PDW waveforms were calculated, for each experiment block. For spatial comparison of the response, a two-dimensional topographical model was constructed. This topography was determined using a cubic interpolation which estimated 10 equally-spaced pixels between the measuring locations highlighted in Fig. 2. The resulting topographical representation was an image consisting of enhanced PDW waveforms and hence was dynamic (i.e., time dependent). To compare the temporal dynamics of the resulting images across the four repetitions of the same musical excerpt, the enhanced PDW waveforms for each image pixel were used to determine a *similarity index* shown in (3).

$$Similarity\;index_{M}^{j,k}=\;\frac{<PDW_{\;M}^{repetition\;j}\left(t\right).\;PDW_{M}^{repetition\;k}\left(t\right)>}{\left\|PDW_{M}^{repetition\;j}\left(t\right)\|*\|PDW_{\;M}^{repetition\;k}\left(t\right)\right\|}$$ (3)

**Fig 3:**
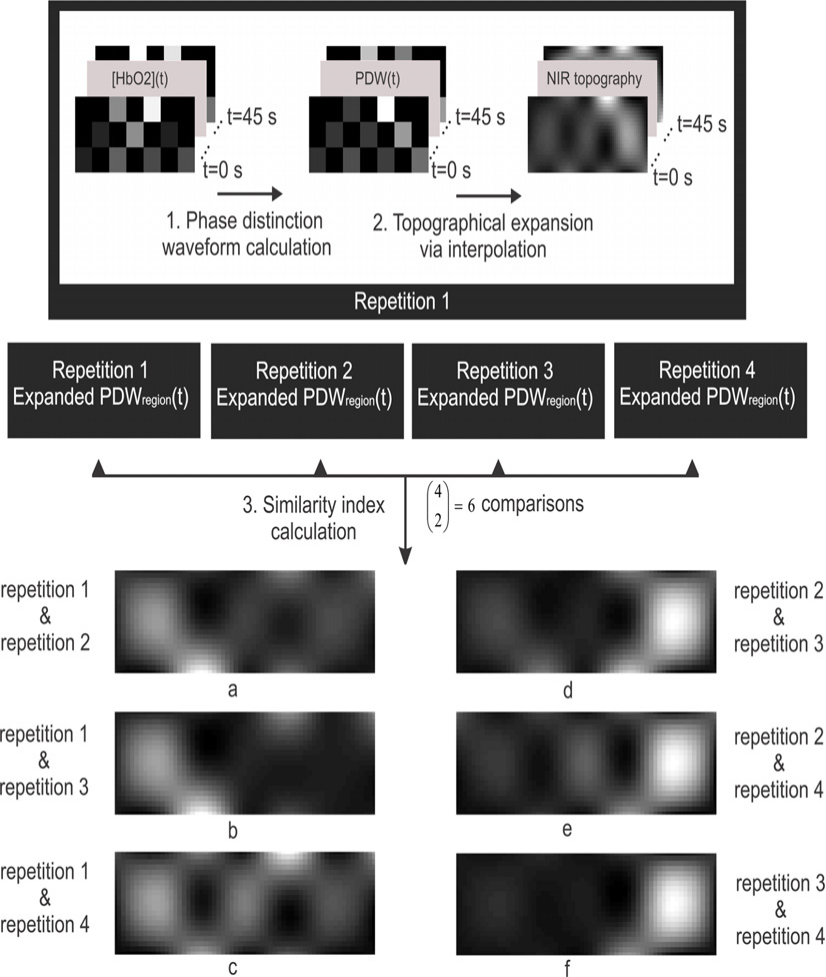
Driving the similarity index. The spatio-temporal characteristics of the hemodynamic response, recorded across regions shown in Fig. 2, were compared across four repetitions of the same musical excerpt. This figure summarizes the procedures involved in comparing these repetitions for one musical excerpt. (NIR: near infrared, PDW: phase distinction waveform (see (2))).

In equation (3), <.> denotes the inner product of the two waveforms, and |.| represents their Frobenius norm. The similarity index is defined for each pixel in the topographical image (represented by M). The *j* and *k* combinations correspond to the repetitions being compared, hence, there are 6 comparisons possible i.e.,$\left(\begin{array}{c} 4\\ 2 \end{array}\right)$. Therefore, the similarity indexes formed 6 images, referred to as similarity topographies. Each of the similarity topographies corresponded to the comparison of two repetitions of the musical excerpt, and represented the spatial and temporal similarities of the measured prefrontal hemodynamics among these repetitions. Fig. 3 depicts the steps involved in determining the similarity topographies.

**Fig 4:**
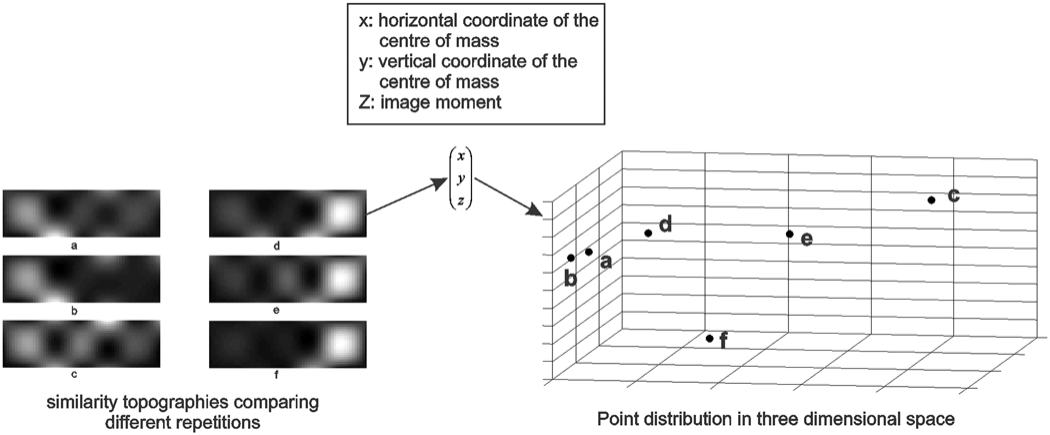
Point representation of similarity topographies. For each musical excerpt, the six similarity topographies were represented using points with (x,y,z) coordinates. The three dimensional distribution of these points are shown for the representative similarity topographies. The distribution of the points in space corresponded to the consistency of the similarity topographies (i.e. points corresponding to similar topographies are closer in the three dimensional space on the right). The point coordinates were normalized to facilitate representation, in this figure.

In order to compare the six similarity topographies, an image moment and the center of mass was determined. For each similarity topography the center of mass was a weighted average of the pixel values with the weights equal to the pixel value. This value represented the distribution of the observed activity across the forehead area. The image moments were determined using Tchebichef polynomials [20]. This method enables compact image representation and has been successfully used for spatial characterization of the prefrontal NIRS in previous studies [21–22]. The order of the image moment was 20. This value was determined empirically by observing the distribution of the moments corresponding to different similarity topographies. Consequently each similar topography was represented by points corresponding to horizontal and vertical coordinates of the center of mass and the moment value. The distribution of these points in the three dimensional space represented the consistency between repetitions of the same musical excerpt. More scattered distributions corresponded to more spatiotemporal variability between repetitions, while more condensed distributions pointed to more consistency among the different repetitions of the musical excerpt. Fig. 4 depicts the steps leading to the three dimensional representation of the similarity topographies.

### Consistency index across all four repetitions

The distribution of the point representation, in the Euclidean space, corresponded to the level of spatiotemporal consistency. Hence, the mean squared error was determined with respect to the average point in space. This value was referred to as the *consistency index* across all four repetitions.

For each of the self-selected songs, six similarity topographies were available for comparison. In order to answer the overarching question of whether repetitions of the same music resulted in similar patterns, a randomly matched set was generated. The generated set was composed of the hemodynamic responses corresponding to four non-identical music excerpts randomly selected from the 60 experimental block pool. For example, the first repetition of song # 1 was matched to the third repetition of song # 3. It is important to note that the randomly matched musical excerpts were not a separate set of excerpts, but they were selected from the same pool of self-selected songs except that they were matched between two non-identical musical excerpts. A total of six randomly matched sets of four were generated for each participant, which were compared to four repetitions of six self-selected songs from the experimental block. The randomly matched sets were subjected to the same analysis depicted in Fig. 3 and Fig. 4, to identify the center of mass and moments of the similarity topographies and ultimately the *consistency index*. The random matching process was repeated 50 times.

### Statistical analysis

The *consistency indexes* calculated for the randomly matched sets were compared with *consistency indexes* calculated for the repeated musical excerpts. For this comparison, Friedman statistic was applied [23]. The six separate songs or randomly matched sets were treated as repeated observations of the ordinal *consistency index* variable.

### Pair-wise consistency indexes

**Fig 5:**
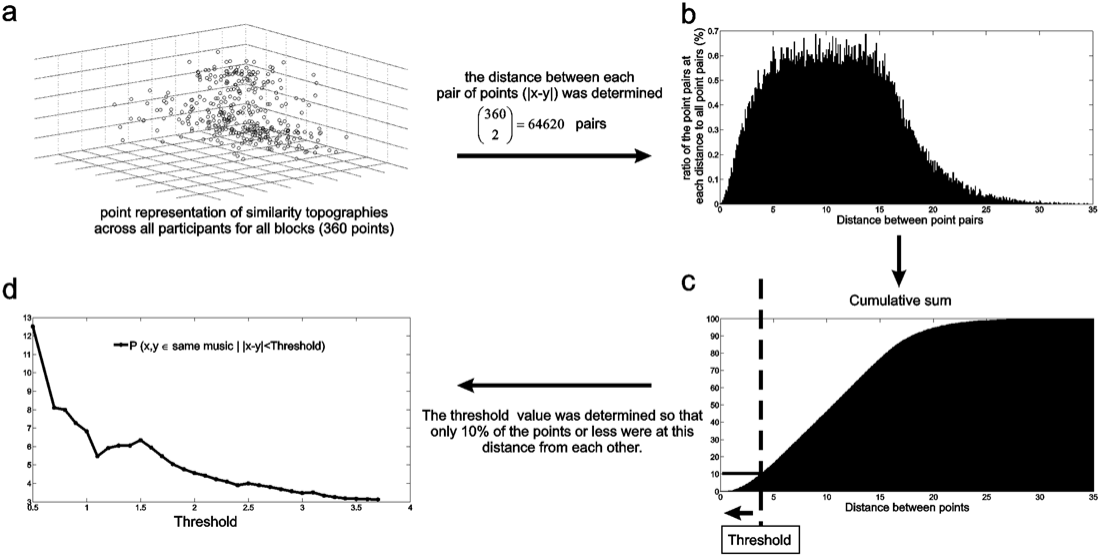
The procedures involved in determining pair-wise consistency indexes. The three dimensional representation of similarity topographies was used to determine distances between points. The histogram and cumulative sum of these distances was used to determine the pair-wise consistency index (i.e. *P*).

In the previous phase, the *consistency index* assessed the consistency between all four repetitions of each musical excerpt, but partial consistency i.e., consistency among a smaller subset of the repetitions of the same musical excerpt, was not investigated by this index. To investigate partial consistency, a *pair-wise consistency index* was defined based on the three dimensional point representations of similarity topographies, shown in Fig. 5. A total of 360 points were available across all participants (six songs, six possible comparisons across ten participants). The *pair-wise index of consistency* was defined based on the distance between each pair of points across 64620 possible distances i.e.,$\left(\begin{array}{c} 360\\ 2 \end{array}\right)$. As shown in Fig. 5b the percent histogram of these distances illustrated the ratio of points falling at each distance from each other, and the cumulative sum of this histogram (Fig. 5c) was used to determine the threshold value. The threshold values were selected so that no more than 10% of the point pairs were within the threshold distance from each other. Ultimately, the probability of the pairs corresponding to the same musical excerpt was estimated given that these points were at a distance less than each threshold value. For example, at a threshold of 3.7 (i.e., 10% cut off shown in Fig. 5c), the ratio of the points belonging to the same musical excerpt at a distance less than 3.7 was determined to all points which were at less than 3.7 distance. The probability estimations were made for a ratio of 1% to 10%, and a step size of 0.5%. For each threshold value, the estimated probabilities (P(x,y∈same musical excerpt |  |x−y|)<threshold) were stored in a vector which was referred to as *P*. The *P* vector represented the ratio of the *pair-wise consistency indexes* for the same musical excerpt, those distances were at lower than *x%* (x = 1, 1.5, 2,…, 10) from each other.

It is important to note that the two analysis methods i.e., consistency index and pair-wise consistency index are proposed to address two different questions. The consistency index compares all four repetitions and the pair-wise consistency index assesses similarity among a subset of trials.

The *P* vector was estimated for all 50 iterations of randomly matched experimental blocks. For each iteration the *P* vector for the randomly matched blocks was compared to *P* vectors corresponding to the experimental blocks. It is important to note that the four musical excerpts in the same group were randomly selected non-identical excerpts. A Kolmogorov-Smirnov test was carried out to assess the significance of any differences observed (p<0.05).

A list of the songs selected by participants is presented in Table 2. Participants had an average of 5.5 years of music training.

**Table 2:** Summary of the music excerpts self-selected by individuals.

Music Title	Composer/Artist
Iris	The Goo Goo Dolls
Tears in heaven	Eric Clapton
Requiem “Dies Irae”	Wolfgang Amadeus Mozart
Untitled	Sigur Rós
Ain’t no mountain high enough	Marvin Gaye and Tammi Terrell
Theme from Schindler’s list	John Williams, “Schindler’s List” motion picture
Julien	Placebo
How to save a life	The Frays
Nocturne No. 20 in C sharp minor	Frédéric Chopin
Virtual insanity	Jamiroquai
Little town	Chorus Beauty and the beast, Page O’Hara and Richard White, “Beauty and the beast” motion picture
A world of our own	The seekers
Veronica Sawyer smokes	Crash Love
Tall trees	Matt Mays and El Torpedo
Cello suite no. 1, prelude	Johann Sebastian Bach
Hallelujah	Jeff Buckley
News bar	Charlie Clouser
Un petit peu d’air	Felipecha
Grand valse brillante	Frédéric Chopin
That’s how you know	Amy Adams, “Enchanted” motion picture
He wasn’t man enough for me	Toni Braxton
Man! I feel like a woman	Shania Twain
Mad world	Gary Jules
Be our guest	Chorus Beauty and the Beast, “Beauty and the Beast”, motion picture
Through and through and through	Joel Plaskett
So close	Jon McLaughlin, “Enchanted” motion picture
Human nature	Michael Jackson
Way over yonder in the minor key	Billy Bragg and Wilco
Every day will be like a holiday	William Bell, (RZA remix)
Give me Jesus	Fernando Ortego
Don’t forget about me	Chris Kirby
I like it	Julio Iglesias
Love you	Free design
Au parc	Chiara Mastroianni
Candle in the wind	Elton John
Red sun	Neil Young
Blower’s daughter	Damien Rice
Bookends	Simon and Garfunkel
All I do is win	DJ Khaled
Hit the road Jack	Ray Charles

The musical excerpt is reported only once, when selected by more than one participant.

## Results

### Spatiotemporal comparison across all six repetitions

**Fig 6:**
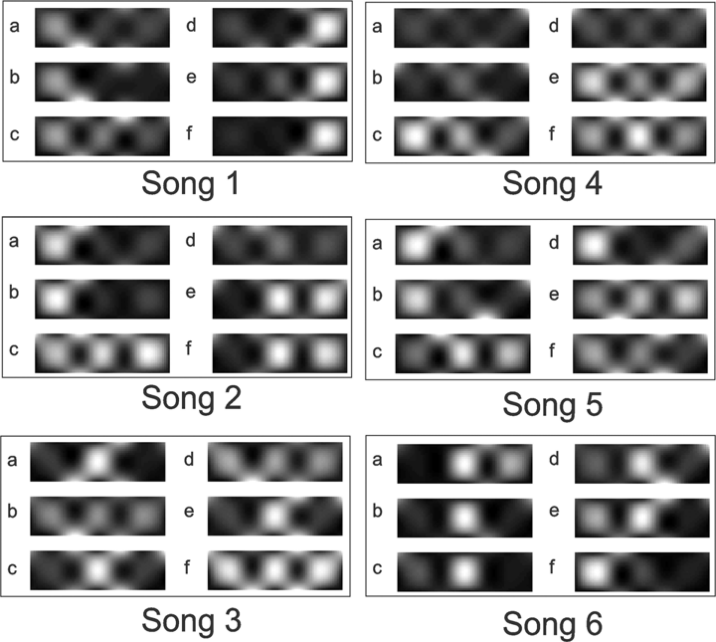
Similarity topographies. The similarity topographies are shown across the recording locations for the six songs selected by participant 1. For each song, the six comparisons are shown for various repetitions, in the same order shown in Fig. 3.

The similarity topographies for participant 1 are shown in Fig. 6. The topographies varied when comparing different repetitions of the same song. For example for song # 2, the topographies *e* and *f* (corresponding to comparisons between 2^nd^ and 4^rd^ repetitions and 3^rd^ and 4^th^ repetitions) are very similar but differ substantially from *b*. Such observations showed the importance of investigating consistency between subsets of the repeated music pieces in addition to all four repetitions.

The difference between the *consistency index* corresponding to repeated musical excerpts and randomly matched sets of non-identical musical excerpts did not reach significance (p<0.05) for any of the 50 iterations. Therefore, no significant difference was observed in the spatiotemporal consistency of the identical self-selected songs and the consistency among exposures to non-identical musical excerpts (i.e. randomly matched sets) when comparing all four repetitions.

### Pair-wise consistency

In the second phase, illustrated in Fig. 5, the similarities were investigated between *pair-wise consistency indexes* (representing similarity topographies). The resulting *P* vectors (P(x,y∈same musical group |  ||x−y||<threshold)) were determined and compared for randomly matched non-identical musical excerpts and identical (repeated) musical excerpts. In 78% of the 50 randomizations, the *pair-wise consistency indexes* were significantly (p<0.05) different for identical musical excerpts compared to non-identical (randomly grouped) musical excerpts. For *pair-wise consistency indexes* that were significantly different, identical musical excerpts resulted in larger *consistency indexes*. This result suggested that when comparing smaller subsets, repeated exposure may result in more consistent spatio-temporal patterns.

### Ratings of emotion

**Fig 7:**
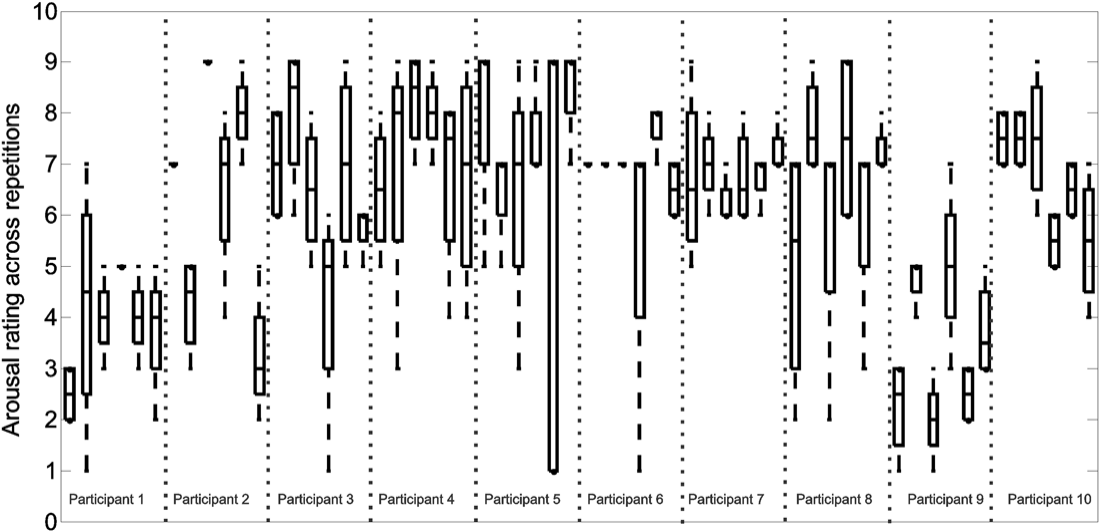
Arousal ratings. The boxplots depict the distribution of arousal ratings (1: least intense, 9: most intense) across four repetitions. For each participant, six songs and hence six boxes are shown.

**Fig 8:**
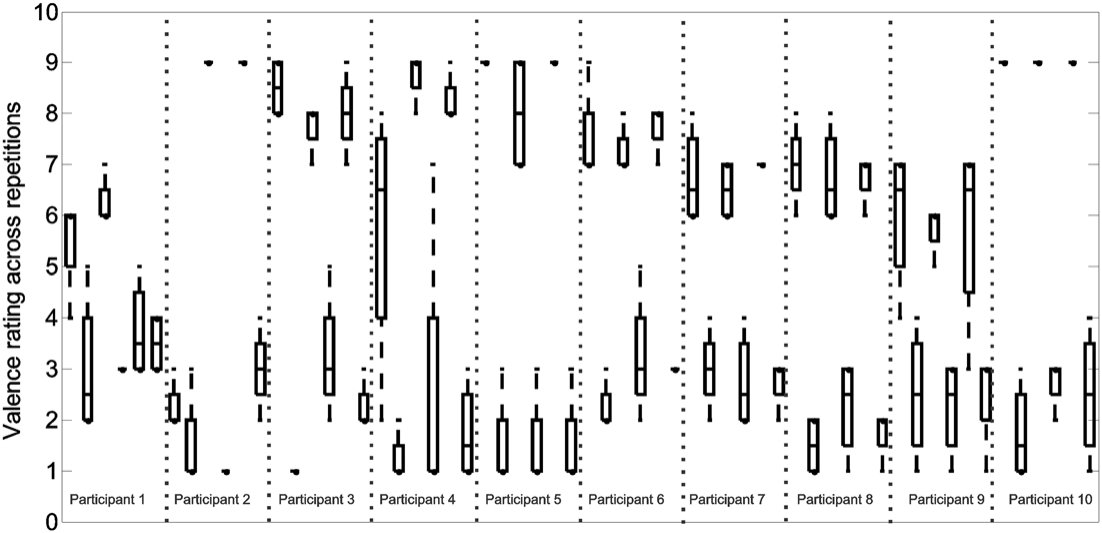
Valence ratings. The boxplots depict the distribution of valence ratings (1: most negative, 9: most positive) across four repetitions. For each participant, six songs and six boxes are illustrated. The self-selected songs represented here are arranged so that the emotional contents are alternating between positive and negative such that the first song is a self-selected piece for inducing positive emotions, the second is a piece inducing negative emotions, and the rest follow similarly.

The distribution of emotional ratings varied across individuals and for various self-selected musical excerpts. Therefore, the emotions experienced during different exposures to the same musical excerpt were not similar. The distribution of the reported ratings of arousal and valence are depicted in boxplots shown in Fig. 7 and Fig. 8, respectively.

## Discussion

The current results were obtained using near-infrared spectroscopy (NIRS) which is suited for prolonged monitoring of prefrontal cortex hemodynamics in naturalistic studies. NIRS is non-invasive, relatively inexpensive, and portable. Therefore, NIRS, which is frequently used in clinical settings for monitoring neonates, children and adults, may be useful in investigating the effect of music as a clinical intervention for these populations. In prolonged studies involving music, repeated exposure to the same musical excerpts may be required. For designing such studies, it is necessary to understand the variability in the prefrontal cortex hemodynamics during repeated exposure to the same musical excerpt, particularly due to the potential influence of music familiarity [10–11]. To measure spatial and temporal hemodynamic variability in this investigation, a *consistency index* was introduced. No significant differences were identified in the hemodynamic response variability (measured using *consistency indexes*) during 4 repetitions of the same musical excerpts, compared to hemodynamic response variability during different musical excerpts. However, the hemodynamic response variability (measured using *consistency indexes*) were significantly different for identical versus non-identical musical excerpts when comparing a subset of repetitions. Therefore, the current results suggest only partial consistency in the observed prefrontal hemodynamic response.

When comparing all four repetitions, the similarity indexes were not significantly different from randomly matched non-identical musical excerpts. However, there was a significant difference between the pair-wise similarity indexes in identical versus randomly-matched non-identical pairs of musical excerpts. This observation indicates that although all repetitions did not result in similar hemodynamic activity patterns, similarities were observed among subsets of these repetitions which were significantly different from randomly matched non-identical musical excerpts.

Previous studies have identified networks specialized for perceiving specific music characteristics [24–25]. These findings suggest that repeated exposure to the same song, with the same characteristics, may induce similar activity patterns in music perception networks which include areas in the auditory perception regions. These networks may also extend to the prefrontal cortex area. For example, using auditory stimuli designed to only vary in harmonic dissonance and unpleasantness, Blood et al. found a significant relationship between subjective ratings of dissonance and orbitofrontal and ventromedial prefrontal cortex activation [26]. These observations support the involvement of prefrontal cortex in the perception of musical characteristics. Therefore, the prefrontal cortex area may show activity modulations in response to dynamic musical characteristics. Janata et al. showed that the rostromedial prefrontal cortex responded to music consonance and more generally to the tonal space [27]. This study involved active listening and identifying musical characteristics in an identical melody modulated in different keys. An important finding of Janata et al. was the dynamic allocation and variability of the recruited brain areas in different exposures [27]. The variability observed in different exposures to the same musical excerpt in the current study, corroborates the findings of Janata et al. [27]. Such variations among repetitions may be attributed to the cognitive and affective mediation that engages the prefrontal cortex area and may vary across different exposures to the same music. In fact, recordings from the prefrontal cortex may tap into three major networks in the brain, salience, central executive and default networks [28]. In addition, medial/lateral prefrontal cortex activity may be influenced by intrinsic cortical hubs [29]. This diversity in the activities engaging the prefrontal cortex may explain the variability in the observed hemodynamic patterns.

Repeated exposures to the same musical excerpt, which shared identical musical characteristics, resulted in different activity patterns in many cases (for an example see Fig. 6). Therefore, music perception was not solely responsible for the activities observed in the measured prefrontal hemodynamics response. Prefrontal hemodynamic activity modulations have previously been observed in response to music with emotional content. In a positron emission tomography study, Blood & Zatorre identified significant changes in the prefrontal cortex hemodynamic response in response to music inducing intense emotional response [30]. The variability observed in the emotional ratings, reported in Fig. 7 and Fig. 8, confirms variations in affective experience in response to the same music, during different exposures to the same song. Variations in the affective response may have influenced the variability in the prefrontal hemodynamic response observed in the current study. To investigate the role of the emotional response in the variability in prefrontal activities observed, future studies should include dynamic emotional rating paradigms. Concurrent emotional rating of music has previously been implemented [31]. However, to the best of our knowledge, brain hemodynamics have not yet been investigated in such studies. Concurrent and dynamic emotional and hemodynamic monitoring may further elucidate the relationship between emotional experience, music, and brain activity.

Recent findings have indicated the possibility of using music as an intervention in clinical settings [9]. Such applications of music may involve repeated exposure to the same piece of music. We embarked on the current investigation to identify whether the physiological changes represented by prefrontal NIRS were similar during different exposures to identical musical excerpts. The results indicated that prefrontal hemodynamics may show similar activity patterns in a subset of the music repetitions, but not in all four repetitions. This observation suggests that when using many repetitions of the same musical excerpt, the prefrontal hemodynamic patterns should not be expected reproducible. Therefore, unlike other clinical interventions such as using a drug to influence a receptor, the results may not be similar in different exposures. Consequently, the analysis methods used in such studies need to be adjusted for the expected variability in response.

Incorporating individual preferences in choosing the studied musical excerpts may be an important factor to consider when using music as a clinical intervention. A recent study by Jiang et al showed a significant reduction in stress among subjects listening to preferred music compared to those who listened to non-preferred music [32]. Based on a number of other studies involving music in clinical settings [9, 33], self-selected musical excerpts were used in this study to accommodate personal preferences. The use of such self-selected musical excerpts, however, introduces variability in musical characteristics, and previous studies have indicated that changes in musical characteristics can change the induced brain activity. For example, using positron emission topography (PET), Blood et al. identified an association between orbitofrontal and frontal polar cortex and decreased musical dissonance [26]. Music with major mode resulted in significant activations in the left medial and superior frontal gyri in an fMRI study conducted by Khalfa et al. [34]. Similar studies have further corroborated the effect of music characteristics on the observed prefrontal hemodynamic response [35–36]. The presence of lyrics is another factor which can influence the observed brain activity. Brattico et al. used fMRI to compare the response to music with and without lyrics and observed significant differences including an increase in the right dorsolateral prefrontal cortex activity in response to music with lyrics compared to instrumental musical excerpts [37]. These findings, call for music characteristic control in music cognition studies. It is not clear whether the variability in music features and genres in the current study has played a role in the observed results. Therefore, music characteristic and genre control is recommended for future studies in order to understand their role in the observed behavioral and functional response.

The sample size is a limitation in this study and may decrease the power to reject the null hypothesis of no difference in favor of the alternative of finding some difference between repetitions of the same musical excerpts. Using the proposed analysis method, future investigations involving more subjects are needed to further evaluate the current results.

Previous studies have shown differences between the observed brain activity in musicians and non-musicians. Such differences may be attributed to variations in music perception due to musical training. Koelsch et al used early right anterior negativity (ERAN) as a marker of musical syntax processing and found increased ERAN amplitude in musicians compared to non-musicians [38]. In another EEG study, Bhattacharya identified a significant increase in the inter-dependencies (a marker of functional relationship) over multiple cortical areas in musicians compared to non-musicians [39]. Subjects participating in the current study varied with respect to their musical training (mean: 5.5 years), but the effect of musical expertise on the observed brain activity variations cannot be investigated due to the small sample size. To address the effect of musical training, future studies are required in which musical expertise is controlled among subjects.

Functional NIRS (fNIRS) experiments with our prefrontal set- up does not provide neuroanatomical resolution specificity of brain fMRI, however, it could allow for recordings that could be extended for hours and many days (and exploring eventually prolonged awake and sleep periods). The light sources and detectors were held in place using a custom-made non-elastic headset which was centered at midline and above the eyebrows in each trial to ensure consistency. However, no information was acquired regarding the underlying neuroanatomical structures. Due to this limitation, we refrained from making any conclusions based on the underlying anatomical structures. Future investigations should consider using a structural imaging modality such as MRI in combination with NIRS to determine the exact neuroanatomical distribution of the observed hemodynamic changes.

## Conclusions

In this study, prefrontal hemodynamic response during repeated exposure to identical musical excerpts was investigated using NIRS technology. For this purpose, a spatiotemporal measure of consistency was introduced. The consistency among four repeated exposures to the same musical excerpt was not significantly different from consistency among non-identical randomly matched musical excerpts, while pair-wise comparisons revealed consistencies which were significantly different from non-identical musical excerpts.

These results highlight the importance of emotional and cognitive processes that may engage prefrontal cortex during different occasions of exposure to identical musical excerpts. Finally, future studies involving dynamic emotional monitoring in addition to prefrontal hemodynamic recording are warranted.

## References

[1] Koelsch S. Towards a neural basis of music-evoked emotions. Trends in cognitive sciences. 2010; 14(3): 131–137. 10.1016/j.tics.2010.01.002 20153242

[2] Trappe HJ. The effect of music on human physiology and pathophysiology. Music and Medicine. 2012; 4(2): 100–105.

[3] Chanda ML, Levitin DJ. The neurochemistry of music. Trends in cognitive sciences. 2013;17(4): 179–193. 10.1016/j.tics.2013.02.007 23541122

[4] Soto D, Funes MJ, Guzmán-García A, Warbrick T, Rotshtein P, Humphreys GW. Pleasant music overcomes the loss of awareness in patients with visual neglect. Proceedings of the National Academy of Sciences. 2009; 106(14): 6011–6016. 10.1073/pnas.0811681106 19307566 PMC2667081

[5] Davis T, Jones P. Music Therapy: Decreasing Anxiety in the Ventilated Patient: A Review of the Literature. Dimensions of Critical Care Nursing. 2012; 31(3): 159–166. 10.1097/DCC.0b013e31824dffc6 22475701

[6] Chan M F, Wong ZY, Thayala NV. The effectiveness of music listening in reducing depressive symptoms in adults: a systematic review. Complementary Therapies in Medicine. 2011; 19(6): 332–348. 10.1016/j.ctim.2011.08.003 22036525

[7] Chan MF, Wong ZY, Onishi H, Thayala NV. Effects of music on depression in older people: a randomised controlled trial. Journal of Clinical Nursing. 2012; 21(5‐6): 776–783. 22035368 10.1111/j.1365-2702.2011.03954.x

[8] Vachiramon V, Sobanko JF, Rattanaumpawan P, Miller CJ. Music Reduces Patient Anxiety During Mohs Surgery: An Open-Label Randomized Controlled Trial. 2013; 39(2): 298–305. 10.1111/dsu.12047 23346989

[9] Chlan LL, Weinert CR, Heiderscheit A, Tracy MF, Skaar DJ, Guttormson JL, et al Effects of patient-directed music intervention on anxiety and sedative exposure in critically ill patients receiving mechanical ventilatory support: a randomized clinical trial. JAMA. 2013; 309(22): 2335–2344. 10.1001/jama.2013.5670 23689789 PMC3683448

[10] Van Den Bosch I, Salimpoor VN, Zatorre RJ. Familiarity mediates the relationship between emotional arousal and pleasure during music listening. Frontiers in human neuroscience. 2013; 7. 10.3389/fnhum.2013.00534 PMC3763198 24046738

[11] Pereira CS, Teixeira J, Figueiredo P, Xavier J, Brattico E. Music and emotions in the brain: familiarity matters. PloS one. 2011; 6(11): e27241 10.1371/journal.pone.0027241 22110619 PMC3217963

[12] Hoshi Y, Huang J, Kohri S, Iguchi Y, Naya M, Okamoto T, et al Recognition of Human Emotions from Cerebral Blood Flow Changes in the Frontal Region: A Study with Event‐Related Near‐Infrared Spectroscopy. Journal of Neuroimaging. 2011; 21(2): e94–e101. 10.1111/j.1552-6569.2009.00454.x 20002968

[13] Moghimi S, Kushki A, Guerguerian AM, Chau T. Characterizing emotional response to music in the prefrontal cortex using near infrared spectroscopy. Neuroscience Letters. 2012; 525(1): 7–11. 10.1016/j.neulet.2012.07.009 22842396

[14] Tai K, Chau T. Single-trial classification of NIRS signals during emotional induction tasks: towards a corporeal machine interface. Journal of NeuroEngineeringand Rehabilitation. 2009; 6(1): 39. 10.1186/1743-0003-6-39 PMC2779792 19900285

[15] Morris JD. Observations: SAM: the Self-Assessment Manikin; an efficient cross-cultural measurement of emotional response. Journal of advertising research. 1995; 35(6): 63–68.

[16] Power S, Kushki A, Chau T. Towards a system-paced NIRS-BCI: differentiating prefrontal activity due to mental arithmetic and mental singing from the no control state. Journal of Neural Engineering. 2011; 8; 066004 10.1088/1741-2560/8/6/066004 21975364

[17] Cope M. The application of near infrared spectroscopy to non invasive monitoring of cerebral oxygenation in the newborn infant. Department of Medical Physics and Bioengineering, University College London. 1992; 214–9.

[18] Cui X, Bray S, Reiss AL. Functional near infrared spectroscopy (NIRS) signal improvement based on negative correlation between oxygenated and deoxygenated hemoglobin dynamics. Neuroimage. 2010; 49(4): 3039–3046. 10.1016/j.neuroimage.2009.11.050 19945536 PMC2818571

[19] Van Drongelen W. Signal Processing for Neuroscientists, A Companion Volume: Advanced Topics, Nonlinear Techniques and Multi-Channel Analysis. Elsevier, Amsterdam 2010.

[20] Zhu H, Liu M, Shu H, Zhang H, Luo L. General form for obtaining discrete orthogonal moments. IET image processing. 2010; 4(5): 335–352.

[21] Schudlo LC. Development of an Optical Brain-Computer Interface using Dynamic Topographical Pattern Classification, Doctoral dissertation, University of Toronto 2012.

[22] Schudlo LC, Power S, Chau T. Dynamic topographical pattern classification of multichannel prefrontal NIRS signals, Journal of Neural Engineering. 2013; 10; 046018. 23867792 10.1088/1741-2560/10/4/046018

[23] Katz MH. Study design and statistical analysis: a practical guide for clinicians. Cambridge University Press 2006.

[24] Warrier CM, Zatorre RJ. Right temporal cortex is critical for utilization of melodic contextual cues in a pitch constancy task. Brain. 2004; 127: 1616–1625. 15128620 10.1093/brain/awh183

[25] Bressler SL, Menon V. Large-scale brain networks in cognition: emerging methods and principles. Trends in cognitive sciences. 2010; 14(6): 277–290. 10.1016/j.tics.2010.04.004 20493761

[26] Blood AJ, Zatorre RJ, Bermudez P, Evans AC. Emotional responses to pleasant and unpleasant music correlate with activity in paralimbic brain regions. Nature neuroscience. 1999; 2(4): 382–387. 10204547 10.1038/7299

[27] Janata P, Birk JL, Van Horn JD, Leman M, Tillmann B, Bharucha JJ. The cortical topography of tonal structures underlying Western music. Science. 2002; 298(5601): 2167–2170. 12481131 10.1126/science.1076262

[28] Sridharan D, Levitin DJ, Menon V. A critical role for the right fronto-insular cortex in switching between central-executive and default-mode networks. Proceedings of the National Academy of Sciences. 2008; 105(34): 12569–12574. 10.1073/pnas.0800005105 18723676 PMC2527952

[29] Buckner RL, Sepulcre J, Talukdar T, Krienen FM, Liu H, Hedden T, et al Cortical hubs revealed by intrinsic functional connectivity: mapping, assessment of stability, and relation to Alzheimer's disease. The Journal of Neuroscience. 2009; 29(6): 1860–1873. 10.1523/JNEUROSCI.5062-08.2009 19211893 PMC2750039

[30] Blood AJ, Zatorre RJ. Intensely pleasurable responses to music correlate with activity in brain regions implicated in reward and emotion. Proceedings of the National Academy of Sciences. 2001; 98(20): 11818–11823. 11573015 10.1073/pnas.191355898 PMC58814

[31] Korhonen MD, Clausi DA, Jernigan ME. Modeling emotional content of music using system identification. Systems, Man, and Cybernetics, Part B: Cybernetics, IEEE Transactions on. 2005; 36(3): 588–599. 16761812 10.1109/tsmcb.2005.862491

[32] Jiang J, Zhou L, Rickson D, Jiang C. The effects of sedative and stimulative music on stress reduction depend on music preference. The Arts in Psychotherapy. 2013; 40: 201–5.

[33] Garza-Villarreal EA, Wilson AD, Vase L, Brattico E, Barrios FA, Jensen TS, et al Music reduces pain and increases functional mobility in fibromyalgia. Frontiers in psychology. 2014; 5. 10.3389/fpsyg.2014.00090 PMC3920463 24575066

[34] Khalfa S, Schon D, Anton JL, Liégeois-Chauvel C. Brain regions involved in the recognition of happiness and sadness in music. Neuroreport. 2005; 16(18): 1981–1984. 16317338 10.1097/00001756-200512190-00002

[35] Green AC, Bærentsen KB, Stodkilde-Jorgensen H, Wallentin M, Roepstorff A, Vuust P. Music in minor activates limbic structures: a relationship with dissonance?. Neuroreport. 2008; 19(7): 711–715. 10.1097/WNR.0b013e3282fd0dd8 18418244

[36] Minati L, Rosazza C, D'Incerti L, Pietrocini E, Valentini L, Scaioli V, et al Functional MRI/event-related potential study of sensory consonance and dissonance in musicians and nonmusicians. Neuroreport. 2009; 20(1): 87–92. 10.1097/WNR.0b013e32831af235 19033878

[37] Brattico E, Alluri V, Bogert B, Jacobsen T, Vartiainen N, Nieminen S, et al Functional MRI study of happy and sad emotions in music with and without lyrics. Frontiers in psychology. 2011; 2. 10.3389/fpsyg.2011.00308 PMC3227856 22144968

[38] Koelsch S, Schmidt BH, Kansok J. Effects of musical expertise on the early right anterior negativity: An event‐related brain potential study. Psychophysiology. 2002; 39(5): 657–663. 12236333 10.1017/S0048577202010508

[39] Bhattacharya J, Petsche H, Pereda E. Interdependencies in the spontaneous EEG while listening to music. International Journal of Psychophysiology. 2001; 42(3): 287–301. 11812395 10.1016/s0167-8760(01)00153-2

